# Diverse effect of WWOX overexpression in HT29 and SW480 colon cancer cell lines

**DOI:** 10.1007/s13277-014-2196-2

**Published:** 2014-06-19

**Authors:** Magdalena Nowakowska, Karolina Pospiech, Urszula Lewandowska, Agnieszka W. Piastowska-Ciesielska, Andrzej Kazimierz Bednarek

**Affiliations:** 1 Department of Molecular Cancerogenesis, Medical University of Lodz, Zeligowskiego 7/9, 90-752 Lodz, Poland; 2 Department of Biomolecular Chemistry, Medical University of Lodz, Mazowiecka 6/8, 92-215 Lodz, Poland; 3 Department of Comparative Endocrinology, Medical University of Lodz, Zeligowskiego 7/9, 90-752 Lodz, Poland

**Keywords:** WWOX, Colon cancer, Microarray, Quantitative RT-PCR

## Abstract

WW-domain-containing oxidoreductase (*WWOX*) is the tumour suppressor gene from the common fragile site FRA16D, whose altered expression has been observed in tumours of various origins. Its suppressive role and influence on basic cellular processes such as proliferation and apoptosis have been confirmed in many in vitro and in vivo studies. Moreover, its protein is thought to take part in the regulation of tissue morphogenesis and cell differentiation. However, its role in colon cancer formation remains unclear. The aim of this study was to characterize the influence of *WWOX* on the process of colon cancerogenesis, the basic features of the cancer cell and its expression profiles. Multiple biological tests, microarray experiments and quantitative reverse transcriptase (RT)-PCR were performed on two colon cancer cell lines, HT29 and SW480, which differ in morphology, expression of differentiation markers, migratory characteristics and metastasis potential and which represent negative (HT29) and low (SW480) *WWOX* expression levels. The cell lines were subjected to retroviral transfection, inducting *WWOX* overexpression. *WWOX* was found to have diverse effects on proliferation, apoptosis and the adhesion potential of modified cell lines. Our observations suggest that in the HT29 colon cancer cell line, increased expression of WWOX may result in the transition of cancer cells into a more normal colon epithelium phenotype, while in SW480, *WWOX* demonstrated well-known tumour suppressor properties. Our results also suggest that *WWOX* does not behave as classical tumour suppressor gene, and its influence on cell functioning is more global and complicated.

**Electronic supplementary material:**

The online version of this article (doi:10.1007/s13277-014-2196-2) contains supplementary material, which is available to authorized users.

## Introduction

Colorectal cancer (CRC) is one of the most common cancers in Western countries. According to American Cancer Society in 2013, it will account for 9 % of new cancer cases and 9 % of tumour-associated deaths, independently on gender [1]. In Poland, CRC has been defined as a tumour of the highest growth rate and was ranked second highest in men and third highest in women in mortality statistics in 2010 [2]. Despite progress in understanding the cancer transformation of colon cells, clinical and histopathological classifications are still commonly used [3].

The majority of CRC cases are sporadic, and three distinct developmental molecular pathways have been described, based on molecular changes within colon epithelial cells. All three depict alterations in genome integrity control and DNA repair mechanisms. The most common is chromosomal instability pathway (CIN) characterized by allelic losses, chromosomal amplifications and mutations within suppressor genes and oncogenes (e.g. *APC*, *KRAS*, *BRAF* and *TP53*). The second pathway, microsatellite instability (MSI), is expressed as frameshift mutations within short, tandemly repeated nucleotide sequences which arise as a consequence of disorders in the DNA mismatch repair system. It mostly not only affects MMR genes such as *MSH2*, *MLH1* and mutS homolog (*MSH*) *6* but also concerns *β*-*catenin*, Bcl-2-associated X protein (*BAX*) and *TGFβIIR* genes. The third pathway, the CpG methylator phenotype (CIMP), involves epigenetic gene silencing through DNA methylation. CIMP cases tend to have v-raf murine sarcoma viral oncogene homolog B (*BRAF*) and Kirsten rat sarcoma viral oncogene homolog (*KRAS*) mutations and rarely, *P53* [4, 5]. It is important to note that colorectal tumours are characterized by different changes at the genomic level characteristic of the particular pathway by which they are derived and reveal diverse clinical features, which are included in the classification created by Jass et al. and Oginio et al. [5–7].

WW-domain-containing oxidoreductase (*WWOX*) is a tumour suppressor gene from the common fragile site FRA16D, whose altered expression has been observed in many tumour types, i.e. breast [8, 9], gastric [10], prostate [11], non-small cell lung cancer [12] and Wilms’ tumours [13]. Its suppressive role and its influence on basic cellular processes such as proliferation and apoptosis have been confirmed in many in vitro and in vivo studies [11, 14–17]. As the WWOX protein is a partner of several transcription factors, it probably also takes part in controlling the expression of genes involved in tissue morphogenesis and cell differentiation [18–20]. Moreover, a correlation between WWOX reduction and clinical factors such as stage, grade and disease progression has been observed in such cases as gastric [10, 21], urinary bladder [22], ovary [23, 24] and breast cancer [25, 26].

However, its role in colon cancer cell transformation is not well defined. In normal colon cells, its expression is quite low, and only individual neuroendocrine cells were found to be positive for WWOX staining, while Goblet cells along the large bowel were negative [27]. In CRC samples, patients without disease recurrence during the observation period were found to demonstrate increased *WWOX* messenger RNA (mRNA) expression, with no correlation with other clinical factors. In the same study, a negative correlation was observed between expression of *WWOX* and cyclin E1, *KI67* and proapoptotic *BAX*/B cell lymphoma 2(*BCl2*) ratio and positive correlation with v-erb-b2 avian erythroblastic leukemia viral oncogene homolog 2 (ERBB) 4 [28]. The last mentioned correlation seems to be intriguing, as it was previously shown that WWOX binds to two cytoplasmic regions of ERBB4 responsible for YAP interactions, and this physical binding results in prevention of ERBB4 translocation to nucleus, which in result may lead to dysregulation of ERBB4 transcriptional activity [29]. In breast cancer samples, the presence of membranous WWOX and ERBB4 strongly correlated with favourable outcome, and such coexpression seemed to have prognostic significance [30].

The aim of this study was to characterize how *WWOX* may be involved in colon cancerogenesis or cancer progression and how it influences the basic cancer cell features (i.e. viability, proliferation and apoptosis) and modifies cell expression profile. The chosen colon cancer cell lines differ with regard to morphology, expression of differentiation markers, migratory characteristics and metastasis potential [31, 32]. Both represent the CIN pathway but differ in ploidy, are MSI stable and overexpress mutated P53 [33].

As HT29 demonstrates negative WWOX expression and SW480 low expression, the cell lines were subjected to retroviral transfection to increase *WWOX* expression.

## Materials and methods

### Cell culture

The HT29 cell line was cultured in McCoy’s 5X Modified Medium (Gibco, Life Technologies) and SW480 in RPMI-1640 (Gibco, Life Technologies). Both media were supplemented with 10 % FBS (Gibco, Life Technologies), 1 % L-glutamine and 1 % *Penicillium*, *Neomycinium* and *Streptomycinium* (PNS; Gibco, Life Technologies).

#### Stable transfection

Cells were transfected with retroviral vector pLNCX2, produced by PT67 packing cells, in two variants: an empty vector (experiment reference) and a vector harbouring *WWOX* complementary DNA (cDNA). A neomycin resistance gene was incorporated in both vectors. In order to increase the effectiveness of transduction, polybrene (Sigma) at a concentration of 8 μg/μl was added to the infection medium. The selection was performed with G418 (Aplichem) at a selective concentration of 400 μg/ml for seven doses every 48 h.

### Biological tests

All biological tests were performed in minimum three independent replicates.

#### Invasion assay

The invasion assay was performed with the BioCoat Matrigel Invasion Chambers, 24-well plate (BD, Immunogen), according to the manufacturer’s protocol. Both variants of examined cells were seeded at a concentration of 1.5 × 10^5^ per chamber. The number of cells which transferred the basic membrane was analyzed with ImageJ 1.34 s software (Wayane Rasband, National Institutes of Health, USA; http://rsb.info.nih.gov/ij/)

#### Growth in soft agar

In order to perform this test, two layers of agarose (Serva) were prepared: a lower layer at a concentration of 0.9 % in full medium and an upper layer at a concentration of 0.3 % in cell suspension. The cells were seeded at a concentration of 1 × 10^4^ cells/ml in each well in a six-well plate. After 23 days of culturing, visualization was preformed with nitro blue tetrazolium (NBT) (Sigma). Colonies were counted with ImageJ 1.34 s software.

#### ECM adhesion

The ability of transducted cell lines to adhere to extracellular matrix (ECM) proteins was assessed using the CytoSelect™ 48-Well Cell Adhesion Assay (Cell Biolabs, Inc) according to the manufacturer’s protocol. The ability of the cells to adhere to fibronectin, collagen I, collagen IV, laminin and fibrinogen was assessed. The concentration of cells used in this experiment was 1 × 10^6^ cells/ml. The absorbance was measured using a BioTek plate reader.

#### Proliferation, viability and apoptosis—triplex assay

A triplex assay was performed measuring the difference between the cell variants with regard to viability, proliferation and apoptosis. All the tests were performed on the same cell culture in order to avoid potential differences in population and culture conditions. Cell viability was assessed using alamarBlue® Cell Viability Reagent (Life Technologies). Data was collected after five 1-h intervals, at a fluorescence excitation wavelength of 550 nm and emission of 590 nm.

A DELFIA® Cell Proliferation Kit (Perkin Elmer) was used to assess the differences in proliferation between the strains while DELFIA DNA Fragmentation Assay (based on TUNEL assay; PerkinElmer) assesses the differences in apoptosis. Both cell lines in both variants were seeded on 96-well plate Wallac Isoplate TC (PerkinElmer) at a concentration of 2 × 10^4^ cells per well. Blank and background wells were also included in the experiment.

The reading of fluorescence for Europium and Samarium was performed with VICTOR X4™ (PerkinElmer) plate reader.

### Statistical analysis of biological tests

The results were presented as means, and the statistical significance was assessed with Student’s *t* test. The confidence level was >95 % (*p* < 0.05).

### RNA isolation and cDNA synthesis

Total RNA was isolated from cell culture using the TRIzol reagent (Life Technologies). Following which, 10 μg of the RNA was reverse transcribed to cDNA in a total volume of 100 μl with ImProm-II reverse transcriptase (RT) (Promega). The reaction conditions were as follows: 5 min incubation at 25 °C, 60 min at 42 °C, heating at 70 °C for 15 min. Finally, 2 μl of cDNA diluted with sterile deionized water was used in the PCR reaction.

The cDNA used in the microarray experiments was subjected to an additional hydrolysis stage (30 min at 65 °C) followed by neutralization and purification. More detailed information on the procedure is available upon request.

### Microarray procedures

Microarray flip dye experiments on cancer cell lines were performed with Human OneArray™ Whole Genome Microarray v 5.1 (Phalanx Biotech) in four replicates. Each sample was hybridized against Universal Human Reference RNA (Stratagene) pooled from ten cancer cell lines. Single-stranded cDNA samples were labelled with Cy3 and Cy5 using ULS™ Labelling Kit (Kreatech Diagnostics, Netherlands).

The preparation of a slide for hybridization included pre-wash in ethanol and pre-hybridization according to manufacturer’s protocol. Hybridization was performed in a humidity chamber filled with 2× SSPE buffer at 42 °C for 16–18 h. Post-hybridization washes were performed with the following buffers: 1× SSPE/0.03 % SDS (2 min, 42 °C), 1× SSPE (2 min, RT) and 0.1× SSPE (rinsed several times, RT).

Scanning and preliminary normalization was carried out with ProScanArray (Perkin Elmer, USA) and ScanArray Express, respectively. Further LOWESS and statistical analysis was carried out with the TM4 software suite provided by The Institute for Genomic Research at http://www.tm4.org/. After modified *T* test analysis, the fold change of differentially expressed genes was calculated, and the Pantherdb online ontology application (www.pantherdb.org) was used to classify them according to ontological terms.

The results were regarded as significant at *p* < 0.05.

### Real-time RT-PCR analysis

Real-time RT-PCR was used to assess the relative expression level of selected molecular markers in order to validate the microarray experiments. A LightCycler 480 II (Roche) was used to perform real-time RT-PCR reactions, which were run in duplicate. PCR products were detected via SYBR Green I and a qPCR Core Kit for SYBR Green I (Eurogentec).

The relative mRNA expressions of genes controlling a variety of cellular processes were analyzed: apoptosis (*BAX*, *BCL2* and *BIRC5*), proliferation and cell cycle (*KI67*, cyclin E1 (*CCNE1*) and cyclin D1 (*CCND1*)), signal transduction (epidermal growth factor receptor (*EGFR*), *ERBB4* *JM-a*) and others (*TP73*, *CDH1*). Real-time RT-PCR was used to assess WWOX transfection efficiency. The mRNA expression level of studied genes was normalized to two reference genes (*RPLP0* and *RPS17*). The calibrator for each reaction was Universal Human Reference RNA (Stratagene). The primers used were intron spanning in order to avoid the amplification of genomic DNA, and detection temperature was designed to avoid non-specific/primer-dimer products. The relative gene expression level was calculated according to Roche algorithm [34].

### Immunoblotting

Total protein was extracted from cells using RIPA protein extraction buffer (50 mM Tris-HCl (pH 8.0), 150 mM NaCl, 0.5 % Na Doc, 0.1 % NP-40, 0.1 % SDS, 2 mM EDTA) with protease, phosphatase inhibitor cocktail and 1 mM PMSF (Sigma-Aldrich, Germany). The Bradford method (Bio-Rad Laboratories) was used for determination of protein concentration, according to the manufacturer’s protocol.

Following this, 10 % SDS-PAGE gel electrophoresis was performed on 60 μg of protein, followed again by PVDF membrane (Sigma-Aldrich, Germany) transfer. Subsequently, the membranes were pre-incubated in 5 % non-fat milk in Tris-buffered saline and Tween 20 (TBST) (20 mM Tris-HCl, 500 mM NaCl, Tween-20, pH 7.5) for 1 h at room temperature and incubated for 19 h at 4 °C in selected primary antibodies (Santa Cruz Biotechnology Inc., USA) at a concentration of 1:100.

Afterwards, TBST washes were performed followed by a 2-h incubation in secondary antibodies conjugated with alkaline phosphatase (Sigma-Aldrich, Germany). Post-incubation washes were repeated in the same conditions as before. Novex® AP Chromogenic Substrate (Invitrogen, USA) was used to induce the colour reaction. Band visualization was performed on the membranes, and ImageJ 1.34 s software (Wayane Rasband, National Institutes of Health, USA, http://rsb.info.nih.gov/ij/) was used for densitometric analysis of protein levels. The results were established as optical density (OD) and normalized to glyceraldehyde-3-phosphate dehyrogenase (GAPDH).

## Results

### Stable transfection confirmation

**Fig. 1:**
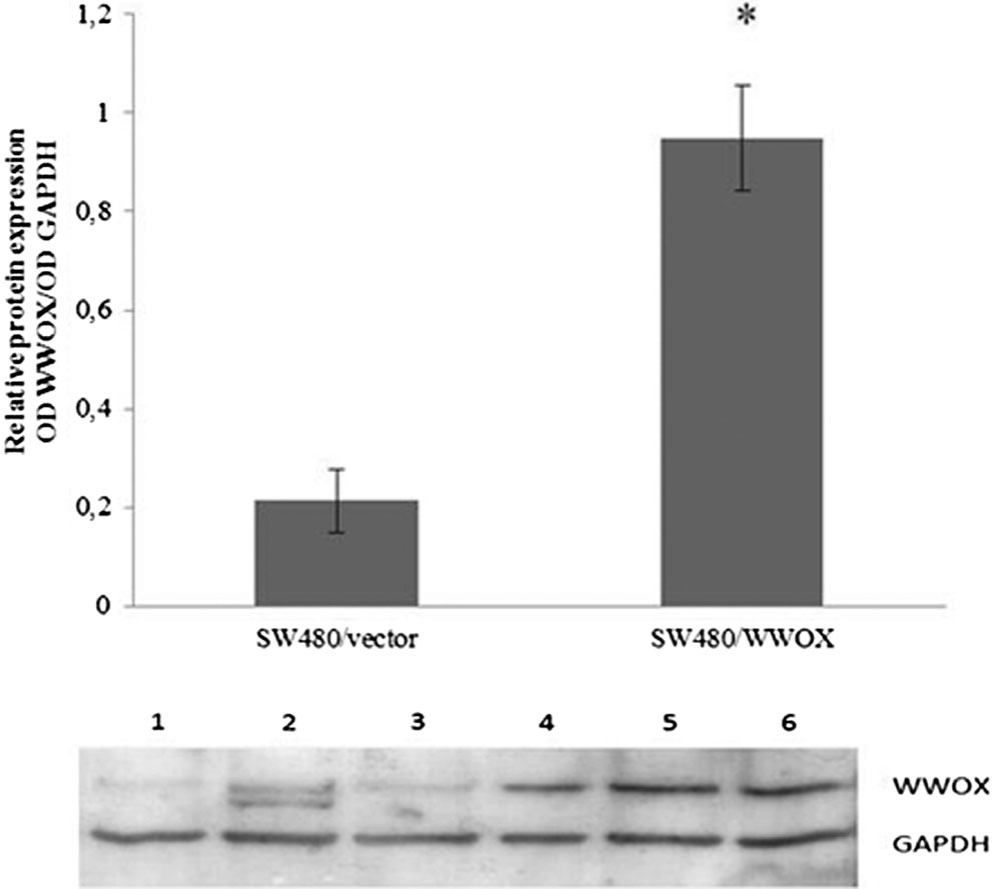
Confirmation of SW480 cell line transfection on the protein level and calculated relative protein expression. The values are mean ± SD. *p < 0.05. *Lines 1*–*3* SW480/vector, *4*–*6* SW480/WWOX

**Fig. 2:**
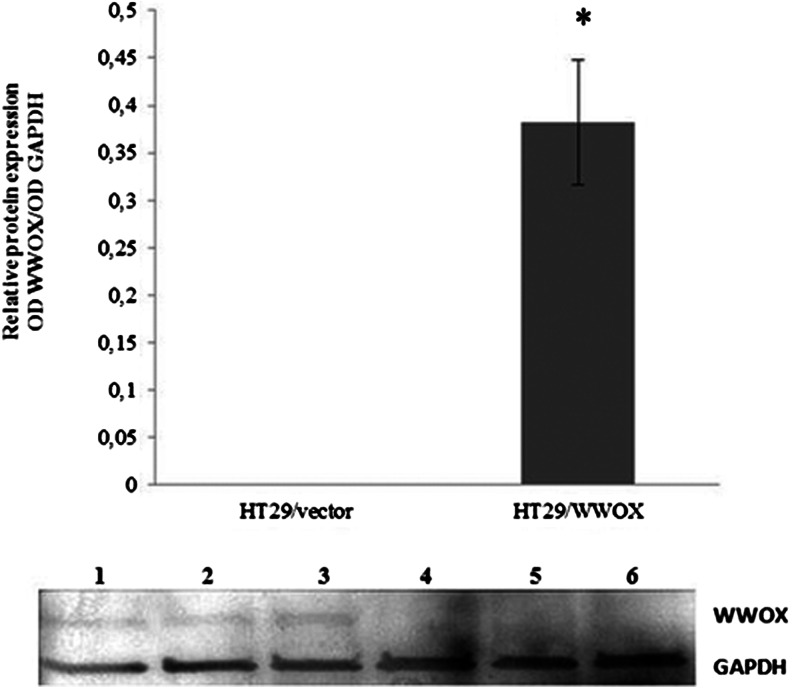
Confirmation of HT29 cell line transfection on the protein level and calculated relative protein expression. The values are mean ± SD. *p < 0.05. *Lines 1*–*3* HT29/WWOX, *4*–*6* HT29/vector

Increased *WWOX* mRNA and protein expression was identified in both colon cancer cell lines (Figs. 1 and 2; Tables 1 and 2). In SW480, the fold change in *WWOX* mRNA expression between transfection variants was more than 137 times, in HT29 more than 37 times. The transfection procedure had no influence on native *WWOX* expression in both cell lines.

**Table 1:** The changes of the relative expression level of cancer-related genes in SW480

	SW480/vector	SW480/*WWOX*	
Gene	Average relative expression level		Regulation+/−
***WWOX***	0.24	32.78	137.42
***KI67***	1.65	0.79	−2.08
***CCNE1***	0.81	0.33	−2.46
***EGFR***	1.29	0.52	−2.49
***HER4 JMa***-***1***	0.16	1.21	7.77
***PTEN***	0.05	0.25	4.92
*CDH1*	0.04	0.05	1.27
*CCND1*	15.62	10.36	−1.51
*TP73*	2.05	1.20	−1.72
*BIRC5*	0.03	0.03	−1.02
*BAX*	0.37	0.38	1.00
*BCL2*	0.02	0.02	1.14

**Table 2:** The changes of the relative expression level of cancer-related genes in HT29 cell lines

	HT29/vector	HT29/*WWOX*	
Gene	Average relative expression level		Regulation +/−
***WWOX***	0.09	3.34	37.82
***BCL2***	0.00051	0.01318	25.64
***HER4***-***JMa***-***1***	0.02	0.44	19.81
***BIRC5***	0.55	0.00	−428.52
***CCND1***	0.23	0.03	−8.41
***KI67***	0.35	0.03	−10.26
***BAX***	1.70	0.29	−5.86
***EGFR***	0.19	0.02	−8.29
***PTEN***	4.37	0.08	−56.72
*CCNE1*	0.20	0.16	−1.26
*CDH1*	5.14	4.99	−1.03
*TP73*	0.19	0.16	−1.15

Bold entries indicate genes with significant change in expression on mRNA level

### Microarray analysis

For both modified colon cancer cell lines, the expression of multiple genes was found to be altered as a result of WWOX overexpression.

The microarray analysis of SW480vector versus SW480/WWOX cell lines revealed over 800 genes which were differentially expressed to a significant degree (*p* < 0.05). The Panther Classification System online ontology application was able to classify over 200 genes into 54 signalling pathways. The remaining genes were marked as unclassified as a result of the complexity of their cell function. The most modified pathways were as follows: wingless-type MMTV integration site family (WNT) (13 genes), integrin signalling pathway (10 genes), inflammation regulated via chemokines and cytokines (10 genes), PDGF pathway (9 genes), cadherin pathway (8 genes) and the transforming growth factor (TGF)-β pathway (7 genes). All identified signalling pathways, with number of genes are available in the sumpelemtary data section. The same ontology program classified received genes according to their function in biological processes, molecular function and cellular component. It is important to note that modifications were identified in the expression of genes regulating the cell cycle of DNA replication, apoptosis and cell motion.

For the HT29 experimental cell line, over 900 genes were differentially expressed to a significant degree (*p* < 0.05). The obtained genes were assigned to 93 signalling pathways. The most highly modified pathways were similar to those found in the SW480 cell line, also included the WNT pathway (14 genes), integrin signalling pathway (12 genes), inflammation regulated via chemokins and cytokins (11 genes), apoptosis (7 genes) and the TGF-β pathway (7 genes). According to other ontology terms, cell cycle and cell motion gene expression were found to be elevated while the expression of the genes responsible for apoptosis was decreased.

Overall, WWOX overexpression resulted in similar genomic modifications in both colon cancer cell lines except for the TGF-β pathway, apoptosis and proliferation. Detailed ontology analysis, for both colon cancer cell lines, is available in Online Resource 1. Microarray data was submitted to GEO Database (reference series: GSE 54301).

### Biological tests

#### Triplex assay

##### Viability

**Fig. 3:**
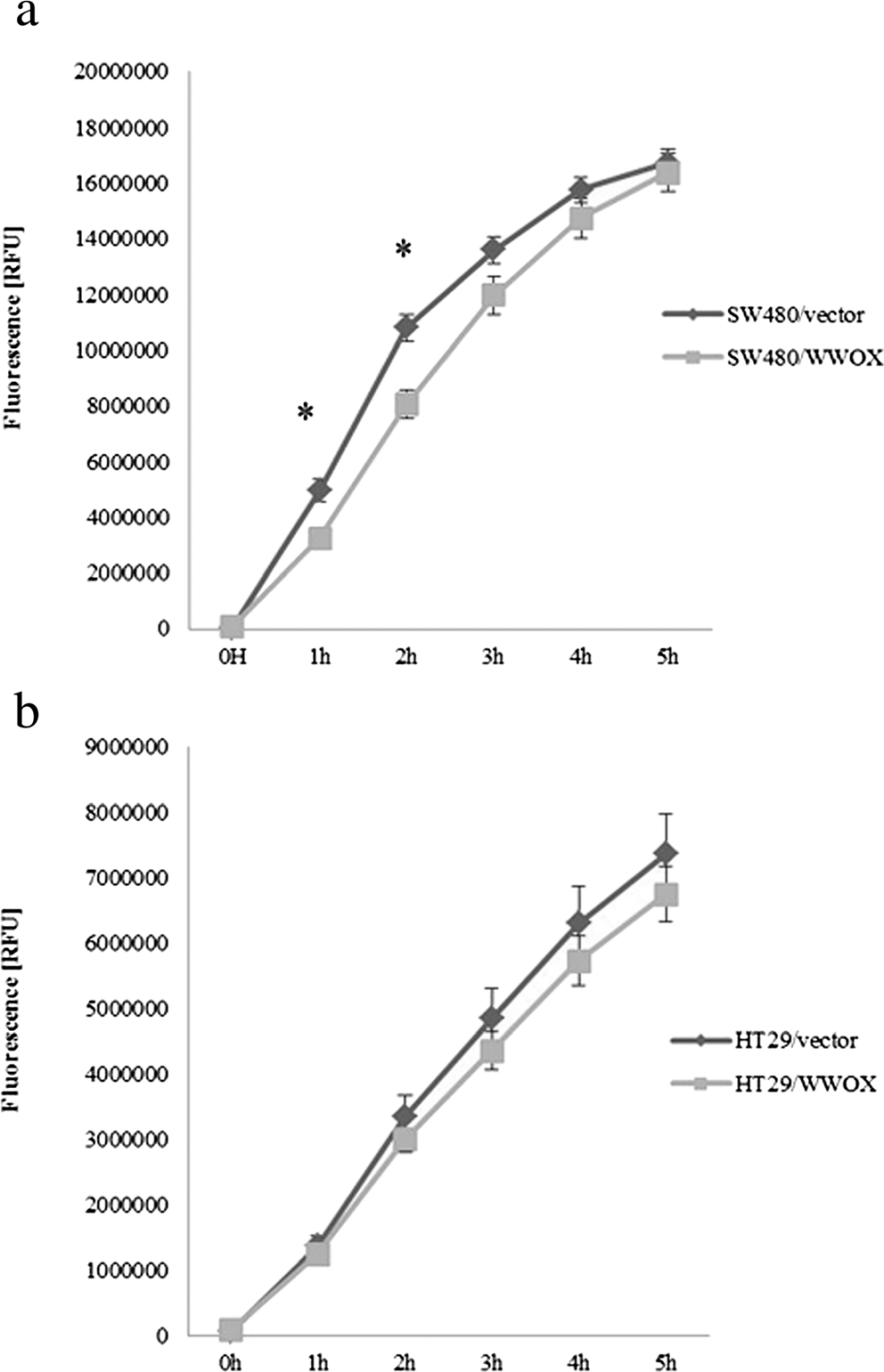
Changes in the viability of **a** SW480 and **b** HT29 cell line variants. Fluorescence is expressed in relative reference units (RFU). The values are mean ± SD. *p < 0.05

Decreased viability was noted for variants overexpressing WWOX in both examined cell lines. However, in SW480/WWOX, the decrease was 20 % in comparison to the SW480 vector and was statistically significant at the first two measurement points. In HT29/WWOX, the decrease was 10 % but insignificant at all measurement points (*p* > 0.05) (Fig. 3a, b).

#### Proliferation and apoptosis

**Fig. 4:**
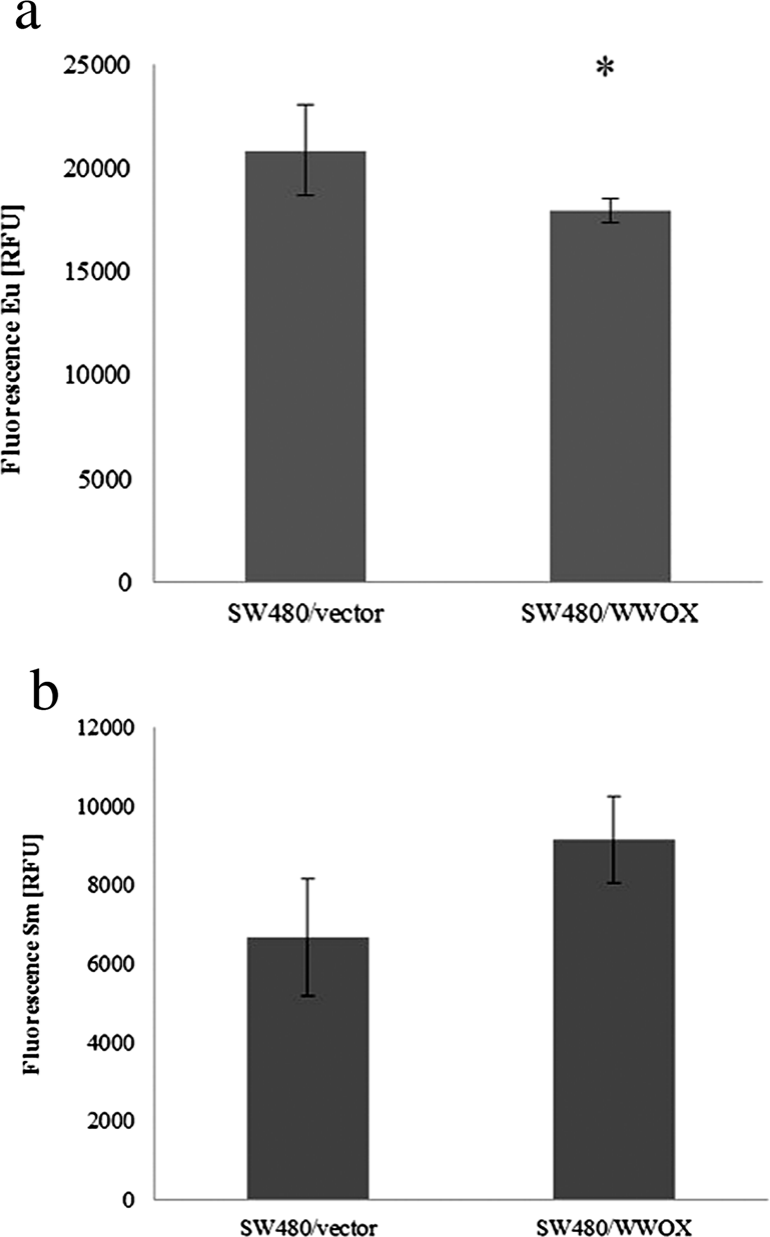
The effect of WWOX overexpression on **a** proliferation and **b** apoptosis in the SW480 cell line. Fluorescence is expressed in relative reference units (RFU). *Eu* europium used as antibody label in proliferation assay, *Sm* samarium used as antibody label in apoptosis assay. The values are mean ± SD. *p < 0.05

For both cell lines, contrasting results were obtained in both tests. In the SW480 cell line, a statistically significant (*p* < 0.05) 20 % decrease in proliferation potential for cell variant harbouring WWOX cDNA was observed in comparison to the experimental reference, the SW480 vector. A 20 % increase of apoptosis was also observed in SW480/WWOX; however, the difference between the examined variants was not statistically significant (*p* > 0.05) (Fig. 4a, b).

**Fig. 5:**
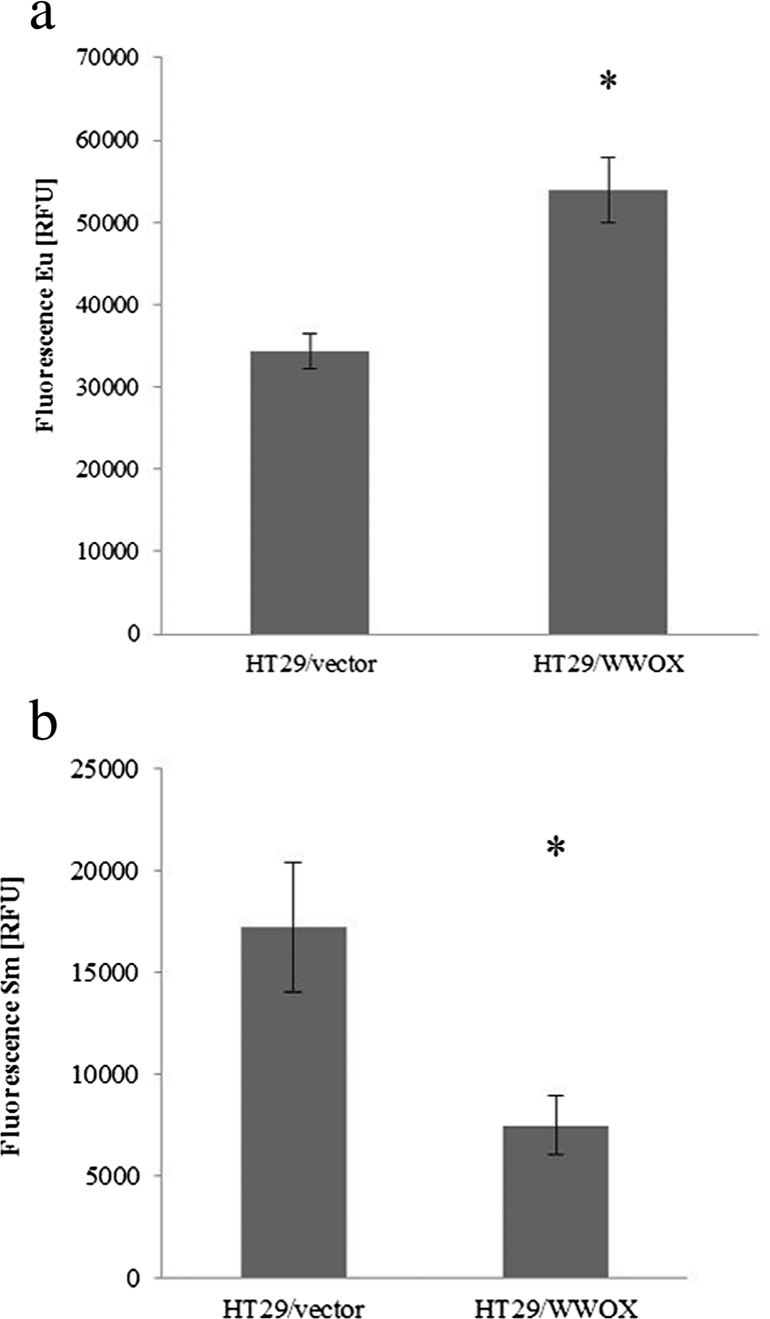
The effect of WWOX overexpression on **a** proliferation and **b** apoptosis in the HT29 cell line. Fluorescence is expressed in relative reference units (RFU). *Eu* europium used as antibody label in proliferation assay, *Sm* samarium used as antibody label in apoptosis assay. The values are mean ± SD. *p < 0.05

In contrast, a 40 % increase in proliferation potential and 60 % decline in apoptosis were seen in a HT29 cell line variant overexpressing WWOX. Both results were statistically significant (*p* < 0.05) (Fig. 5a, b)

#### ECM adhesion

**Fig. 6:**
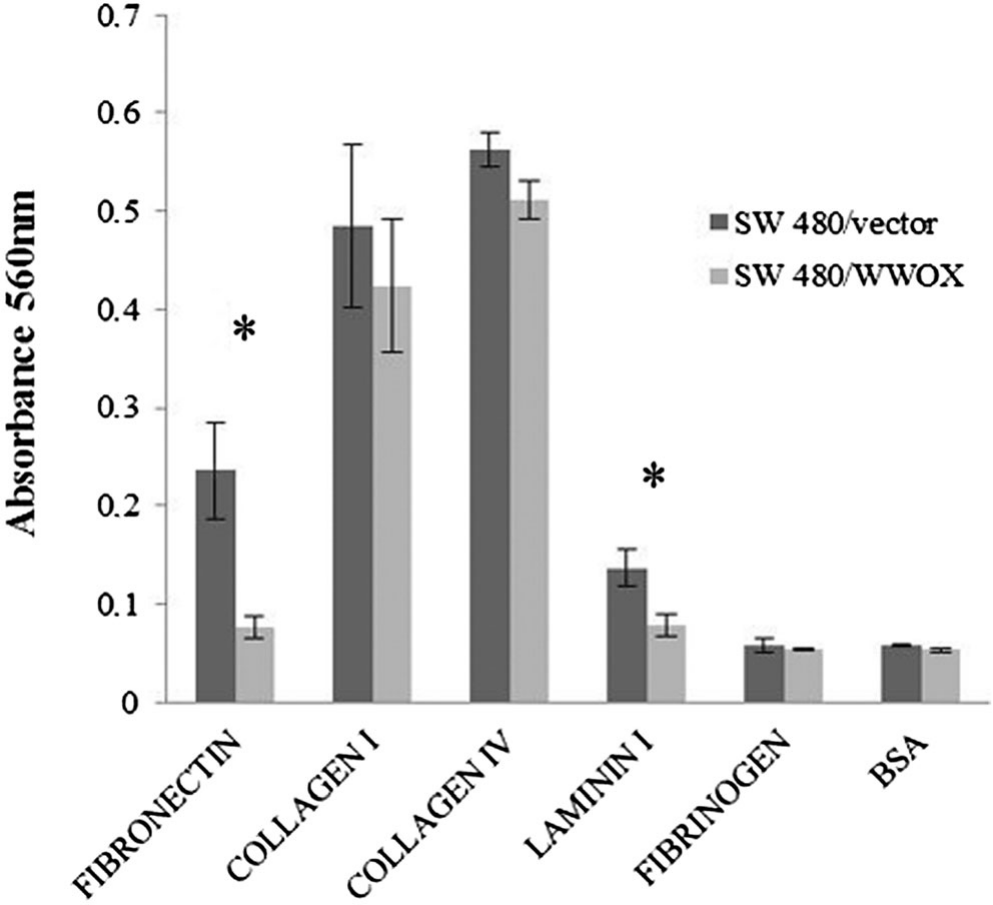
Adhesion to ECM proteins for the SW480 transfected cell line. The values are mean ± SD. *p < 0.05

Statistically significant (*p* < 0.05) decreases in SW480/WWOX cell line adhesion was observed: Adhesion to fibronectin dropped to a third, while adhesion to laminin I halved. The process of adhesion to the rest of the examined ECM proteins did not reveal any differences between the two variants of the SW480 colon cancer cell line (Fig. 6).

**Fig. 7:**
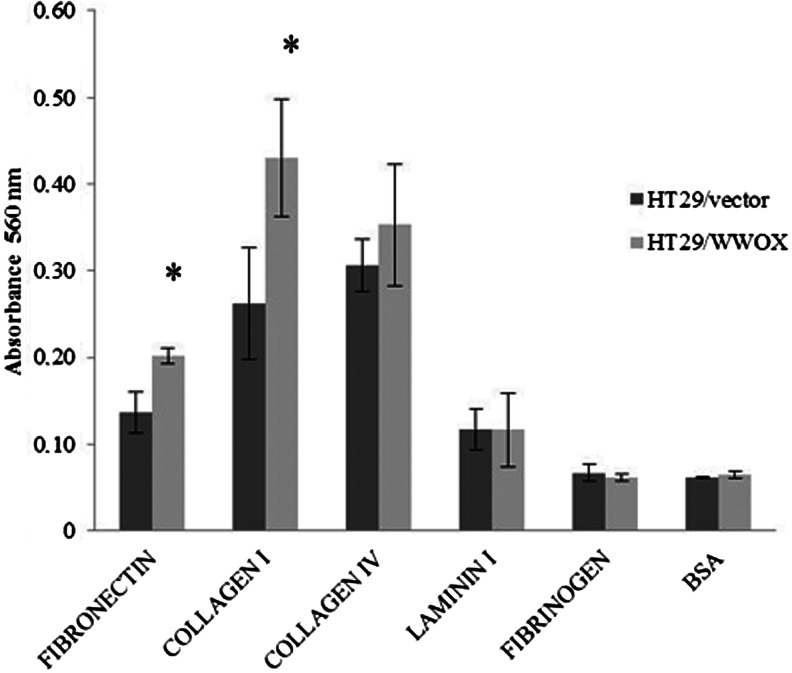
Adhesion to ECM proteins for the HT29 transfected cell line. The values are mean ± SD. *p < 0.05

In contrast, the HT29/WWOX cell line variant, in comparison to the HT29/vector, demonstrated a 2-fold increase in adhesion to collagen I and an increase in adhesion to fibronectin by more than a third. Both results were statistically significant (*p* < 0.05). There were no other differences in adhesion between HT29 WWOX/HT29 vectors to other examined ECM proteins (Fig. 7).

#### Invasion assay

**Fig. 8:**
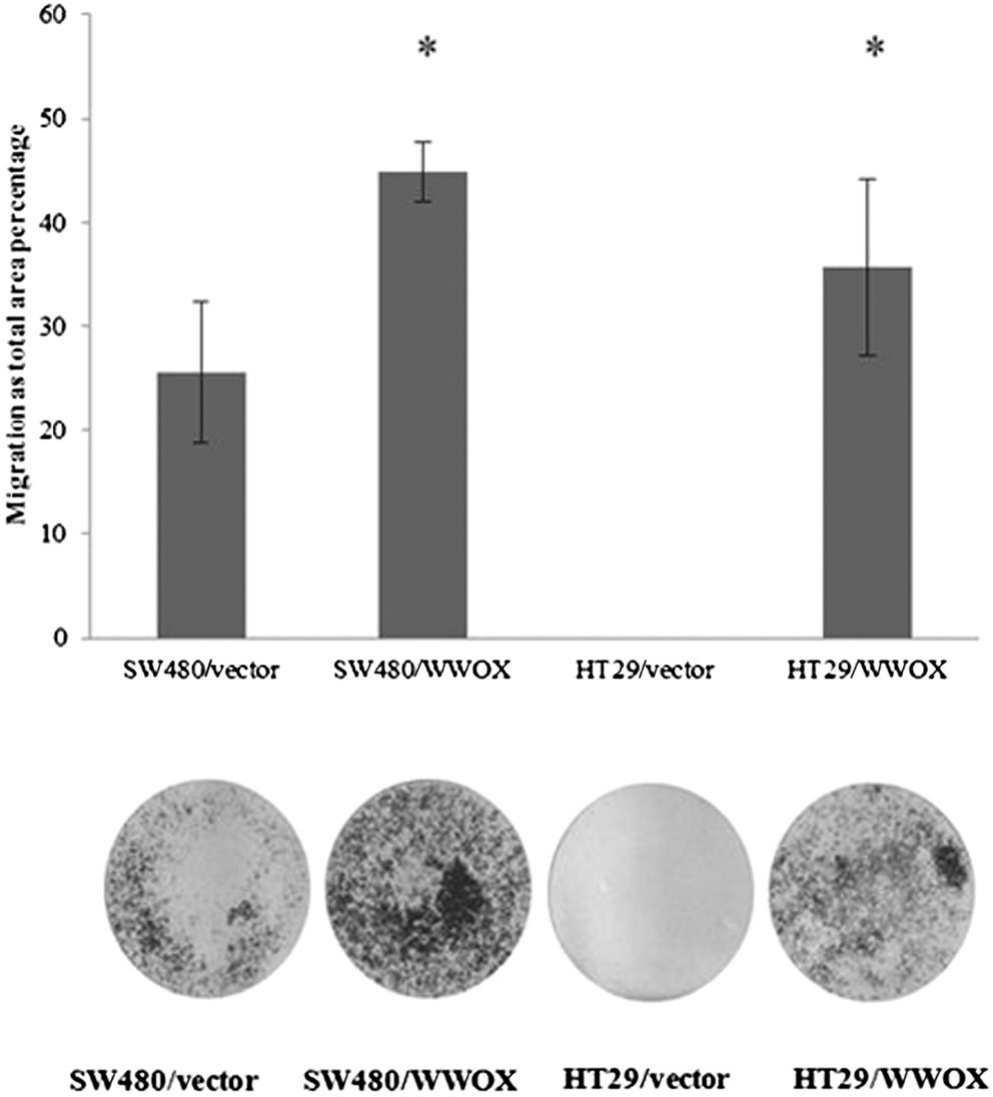
Invasion assay and calculated difference in migration for both transfected colon cancer cell lines. The values are mean ± SD. *p < 0.05

Statistically significant (*p* < 0.05) changes were seen in invasion for both cell lines of variants overexpressing WWOX. In SW480/WWOX, invasion was 50 % higher than the level measured in the SW480 vector, i.e. the variant without WWOX cDNA. Regarding the other colon cancer cell line, the ability to invade the basal membrane was seen in the HT29/WWOX cell variant (Fig. 8).

#### Growth in soft agar

**Fig. 9:**
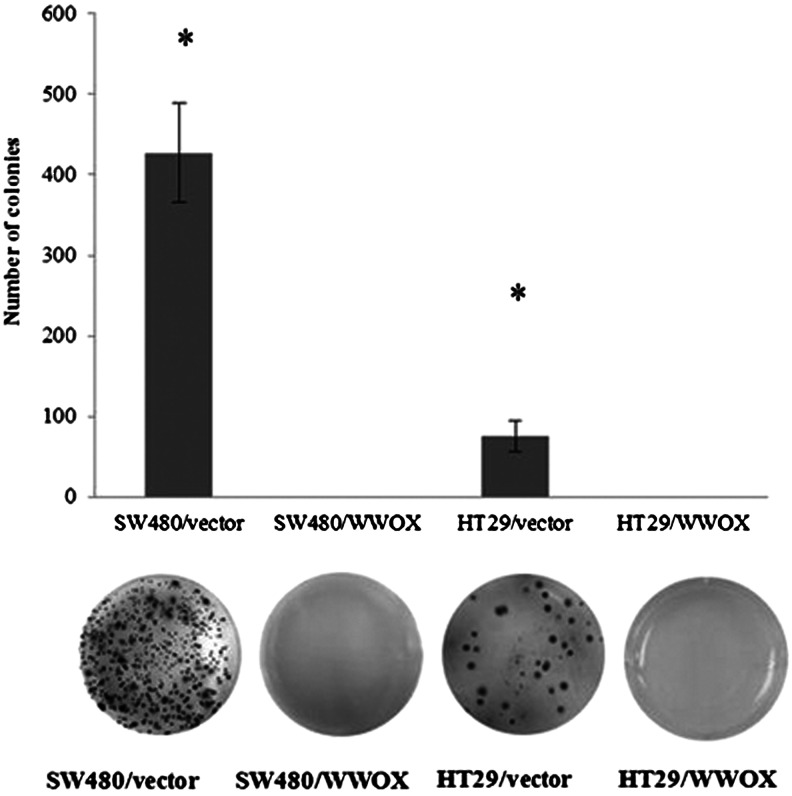
Growth in soft agar and calculated number of colonies for both cell lines. The values are mean ± SD. *p < 0.05

Complete inhibition of growth in suspension was seen in both cell lines overexpressing the WWOX gene (*p* < 0.05) (Fig. 9).

### Real-time RT-PCR

In the SW480/WWOX colon cancer cell line, the expression of genes regulating the cell cycle (*CCNE1*, *KI67*) and signal transduction (*EGFR*) were found to be significantly downregulated, in contrast to the expression of *ERBB4 JMa*-*1* and the suppressor gene *PTEN*. More detailed information, i.e. mean gene mRNA expression and calculated fold change for each gene between cell line variants, is presented in Table 1.

In the other colon cancer cell line, mRNA expression of *BCL2* and *ERBB4 JMa*-*1* was seen to be upregulated in the presence of WWOX. The remaining examined genes were downregulated in HT29/WWOX in comparison to the HT29 vector cell line variant. Detailed information is presented in Table 2.

### Microarray validation

**Fig. 10:**
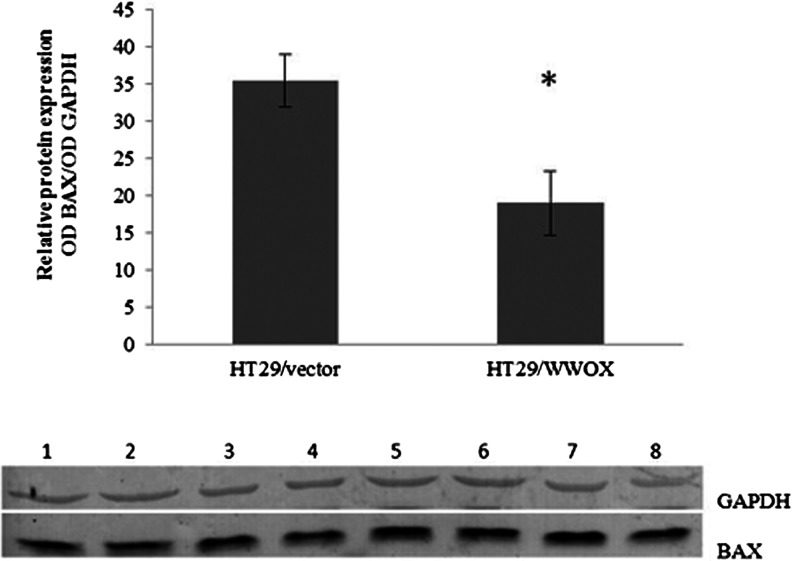
Validation of microarray experiments. The values are mean ± SD;*p < 0.05; *Lines 1*–*4* HT29/vector, *5*–*8* HT29/WWOX

The mRNA expression levels of the *BAX* and oestrogen receptor (*ESR*) *2* genes, whose expression was altered in the HT29 cell line, were analyzed with real-time RT-PCR. The changes in the level of expression of selected genes in microarray analysis positively correlated with the real-time RT-PCR results. The results obtained from both techniques confirmed similar trends of gene expression profiles, i.e. downregulation of *BAX* (the fold change in expression was about 1.5 times in microarray data and 5-fold decrease in RT-PCR) and upregulation of *ESR2* (1.24 upregulation in microarray data, 10-fold in real-time RT-PCR; the relative expression values were 0.105 vs 1.16 for HT29 vector vs HT29/WWOX, respectively). The downregulation of BAX was also noticed on the protein level (1.9-fold decrease) (Table 2, Fig. 10).

## Discussion

The function of *WWOX* in the suppression of cancerogenesis has been confirmed previously in cancers of hormone-related tissues, including breast, ovary and prostate [9, 11, 17, 23, 35], but also in cancers from gastrointestinal tract, such as gastric [10], oesophageal [36], pancreatic [14] and liver tissue [37]. This suppressive effect has been mainly assigned to inhibition of the proliferation and induction of apoptosis. In our present study, we observed *WWOX*-dependent changes in proliferation and apoptosis in two colon cancer cell lines HT29 and SW480, which vary in terms of epithelial-mesenchymal transition (EMT) markers, WNT activity and stemness signatures. SW480 has a high EMT markers expression, with a migratory capacity phenotype and gene expression similar to those of a microdissected tumour epithelium and is a good model of an invasive tumour while the HT29 cell line has been ranked lower in terms of EMT and humanized intestinal stem cell signature (HuISC) signatures, and its expression profile is the closest to the gene expression profile of bulk tumour tissue cells. The HT29 cell line seems to be most similar to small intestine enterocytes and, in specific culture conditions, is able to show distinct differentiation similar to the enterocyte pathway [38]. Our experiments seem to confirm and delineate further behavioural differences between HT29 and SW480 and indicate the diverse effects of WWOX functioning in colon cancerogenesis.

SW480 overexpressing WWOX showed a statistically significant inhibition of proliferation (*p* = 0.04) and a strong tendency towards initiation of apoptosis (*p* = 0.07), which is consistent with previous results observed in breast cancer cell lines (BT-474, MDA-MB-231, HCC1937) [17], lung cancer cell lines (A549, H460, H1299) [39, 40], ovarian cancer line (A2780) [41], liver cancer (SMMC-7721) [16] and other cell lines [42]. The results observed for the SW480 cell line, as for other mentioned cancer cell lines, seem to be an effect of *WWOX* restoration, as SW480 has moderately low WWOX expression (relative expression 0.23).

The microarray data presented herein reveals changes in the expression of genes regulating apoptosis and proliferation, which have been confirmed by real-time RT-PCR to be associated with a significant downregulation of genes connected with cell cycle and proliferation (*CCND1*, *CCNE1* and *KI67*). However, no WWOX-induced changes were observed in the transcription of apoptosis-related genes (*BAX*, *BCL2*, *BIRC5*), whose expression was observed to correlate with *WWOX* in colon tumour specimens [28] and other cancer tissues [13, 26, 43].

Contrary to the SW480, the results of the HT29 biological tests indicated a statistically significant upregulation of proliferation and apoptosis inhibition. This discrepancy between colon cancer cell lines seems to reflect the changes on the transcriptome level indicated by microarray experiments and real-time RT-PCR. Although real-time RT-PCR analysis revealed decreased expression of *CCND1*, *CCNE1* and *KI67* in HT29/WWOX, microarray analysis identified more than 53 genes which play a role in cell cycle regulation, with the overall effect being proliferation induction. Among the genes assigned to regulate proliferation, *ESR2* merits special attention, whose increased expression was confirmed by real-time RT-PCR. Previously, only oestrogen receptor alpha expression had been found to correlate with WWOX expression [26, 44]. However, *ESR2* gene expression is characteristic feature of a normal colon epithelium, and it is downregulated in CRC; in addition, its decreased expression has been associated with Duke’s stage [45], and, according to Konstantinopoulos et al., the loss of ESR2 expression is associated with the disability of cancer cells to differentiate [46]. Hence, its elevated expression, associated with *WWOX* gene overexpression, may result in a higher cell proliferation/differentiation potential.

In addition, the discrepancy between SW480 and HT29 regarding the effect of WWOX on proliferation and apoptosis may also be explained by the diverse effect of WWOX on the TGF-β pathway revealed by the microarray study. The TGF-β pathway is a major regulator of proliferation, apoptosis, cell differentiation and ECM remodelling. However, its function in cancerogenesis is ambiguous. In a normal colon epithelium and in the early stages of cancer formation, this pathway induces apoptosis and inhibits tumour growth. In advanced stages, it loses its suppressive function, leading to tumour progression [47, 48]. Previously, it has been shown that upon pathway initiation, the TGF-β1 protein binds to membranous Hyal-2 and, as a result, forms a complex with WWOX. This complex relocates to the nucleus where the activation of mothers against decapentaplegic homolog (SMAD)-driven promoters is thought to be controlled, thus regulating cell death and growth [49].

In the SW480 cell line with ectopically increased WWOX expression, we observed downregulation of genes belonging to the canonical TGF pathway (i.e. SMAD1 and SMAD2), while an increase of SMAD2 and two growth differentiation factors (GDF), i.e. GDF11 and GDF6, was observed in HT29. These factors are bone morphogenetic proteins (BMPs), which are secreted in stromal cells and induce colon cell differentiation, constituting an alternative TGF-β pathway. Although their highest expression was observed in the upper parts of the colon tops, they are also expressed in the basal crypts, albeit in an inactive state [50, 51]. GDF11 is known to play a role in colon cancerogenesis, and it has been indicated that it may influence the proliferation and differentiation of stem cells [52].

The overall results obtained for the HT29 cell line may reflect alterations of the TGF-β pathway, which redirects HT29 cells overexpressing *WWOX* towards the phenotype of a normal colon cell from the upper parts of the colon crypts. This observation is consistent with the description of the character of the HT29 cell line, insofar that they demonstrate many biochemical and physiological features of normal colorectal epithelial cells [53], which may be further enhanced via *WWOX* overexpression.

In our study, the transfected cell lines were found to have different adhesion properties to ECM proteins. SW480/WWOX demonstrated decreased adhesion to fibronectin, consistent with previous findings for ovarian cancer cell lines PEO1, SCOV3 [35] and laminin, which has been identified as a predictive factor for tumour progression [54], whereas HT29/WWOX demonstrated increased adhesion to fibronectin and collagen I. These differences imply that diverse adhesion may reflect the process of cell remodelling of HT29.

The diverse effect of WWOX observed in these two colon cancer cell lines may also reflect the different stage of cancerogenesis progression. In this case, the transcription mechanisms may react in different ways to changes in WWOX protein level.

However, the influence WWOX on the aggressiveness of cancer cells, measured as growth in soft agar, seems to be identical for both experimental colon cancer cell lines and consistent with previous findings [11, 14, 15, 19] insofar that a statistically significant inhibition of colony formation was noted in both cell lines overexpressing WWOX. Regard the invasion assay, the same results was observed for both cell lines. As in the MDA-MB 231 breast cancer cell line, increased expression of WWOX resulted in increased invasion through basal membrane, which may be connected with WWOX regulation of cell motility, and may perhaps exert an influence on tissue remodelling [19].

## Conclusions

The study investigated the role of the *WWOX* gene in colon cancerogenesis. We have noticed reduction of proliferation rate and adhesion to ECM proteins in SW480 colon cancer cell line overexpressing WWOX, which observations seem to confirm the well-described tumour suppressor properties of *WWOX* gene. In the other experimental colon cancer cell line HT29, increased expression of investigated gene resulted in the opposite effect on apoptosis, proliferation and adhesion seen in SW480 cell line. However, taking under consideration cell line features, our observations suggest that increased expression of WWOX may transform HT29 cancer cells into a more normal colon epithelium phenotype. Our results also suggest that WWOX does not behave as classical tumour suppressor gene and its influence on cell functioning is more global and complicated. Our observations might be also an effect of chromosomal instability and mutated p53 in both cell lines, which generate high genomic complexity in which WWOX might be also involved. Further studies ought to be conducted in order to specify the role of the WW-domain-containing oxidoreductase gene in the process of colon cancer formation.

## Electronic supplementary material

Below is the link to the electronic supplementary material.

ESM 1 (PDF 32 kb)
