# Supplementary material for: Diverse effect of WWOX overexpression in HT29 and SW480 colon cancer cell lines
Source: Tumour Biol. 2014 Jun 19;35(9):9291–301. doi: 10.1007/s13277-014-2196-2 (PMC4190457; doi:10.1007/s13277-014-2196-2)
Supplement: Supplementary file 1 — (PDF 32 kb) [file 13277_2014_2196_MOESM1_ESM.pdf]

Diverse effect of WWOX overexpression in HT29 and SW480 colon cancer cell lines

Magdalena Nowakowska[1*], Karolina Pospiech[1], Urszula Lewandowska[2],Agnieszka W. Piastowska-Ciesielska[3], Andrzej Kazimierz Bednarek[1]

[1] Department of Molecular Cancerogenesis, Medical University of Lodz, Zeligowskiego 7/9

90-752 Lodz, Poland

[2] Department of Biomolecular Chemistry, Medical University of Lodz, Mazowiecka 6/8, 92-215 Lodz, Poland

[3]Department of Comparative Endocrinology, Medical University of Lodz, Zeligowskiego 7/9

90-752 Lodz, Poland

[*]Corresponding author

magdalena.nowakowska@umed.lodz.pl

Department of Molecular Cancerogenesis

Medical University of Lodz

Zeligowskiego 7/9

90-752 Lodz, Poland

Tel. +48 42 6393180

In this file we present ontology analysis of microarray data performed with the on line ontology application Pantherdb (www.pantherdb.org) for both experimental cell lines.

All attached tables are presenting the specific ontology term and number of genes which were included.

Table 1 The ontological analysis of the genes modulated by the *WWOX* overexpression in HT29 cancer cell line. Pathways

Pathway	Number of genes
Unclassified	729
Wnt signaling pathway	14
Integrin signalling pathway	12
Inflammation mediated by chemokine and cytokine signaling pathway	11
Heterotrimeric G-protein signaling pathway-Gq alpha and Go alpha mediated pathway	11
Heterotrimeric G-protein signaling pathway-Gi alpha and Gs alpha mediated pathway	11
Angiogenesis	9
Huntington disease	9
Alzheimer disease-amyloid secretase pathway	8
Apoptosis signaling pathway	7
TGF-beta signaling pathway	7
p53 pathway	6
PDGF signaling pathway	6
VEGF signaling pathway	5
Metabotropic glutamate receptor group III pathway	5
Endothelin signaling pathway	5
Cadherin signaling pathway	5

Table 2 The ontological analysis of the genes modulated by the *WWOX* overexpression in HT29 cancer cell line. Molecular Function.

Molecular Function	Number of genes
Unclassified	323
binding	273
catalytic activity	208
nucleic acid binding	151
protein binding	122
hydrolase activity	99
DNA binding	94
transcription factor activity	83
transcription regulator activity	83
receptor activity	70
transferase activity	57
structural molecule activity	53
enzyme regulator activity	49
transporter activity	49
transmembrane transporter activity	48
receptor binding	43
peptidase activity	35

structural constituent of cytoskeleton	33
hydrolase activity, acting on ester bonds	27
kinase activity	25
oxidoreductase activity	24
cation transmembrane transporter activity	23
ion channel activity	21
G-protein coupled receptor activity	21
small GTPase regulator activity	20
protein kinase activity	19
RNA binding	19
enzyme inhibitor activity	18
calcium ion binding	17
ligase activity	17
GTPase activity	15
transcription cofactor activity	15
serine-type peptidase activity	13
cytoskeletal protein binding	12
peptidase inhibitor activity	11
kinase regulator activity	10
ubiquitin-protein ligase activity	10
phosphatase activity	10
structural constituent of ribosome	10
voltage-gated ion channel activity	10

Table 3 The ontological analysis of the genes modulated by the *WWOX* overexpression in HT29 cancer cell line. Cellular component

**Cellular Component**	**Number of genes**
Unclassified	767
intracellular	41
cytoskeleton	33
extracellular matrix	23
extracellular region	23
actin cytoskeleton	15
microtubule	10
protein complex	8
cytoplasm	8
organelle	7
mitochondrion	6
mitochondrial inner membrane	5
SNARE complex	5
ribonucleoprotein complex	5
intermediate filament cytoskeleton	4
cell junction	4
plasma membrane	4

Table 4 The ontological analysis of the genes modulated by the *WWOX* overexpression in HT29 cancer cell line. Biological Process

**Biological Process**	**Number of genes**
metabolic process	319
primary metabolic process	298
Unclassified	272
cellular process	245
cell communication	168
signal transduction	159
nucleobase, nucleoside, nucleotide and nucleic acid metabolic process	138
transport	123
protein metabolic process	113
developmental process	95
immune system process	85
transcription from RNA polymerase II promoter	81
transcription	81
cell surface receptor linked signal transduction	75
system process	68
regulation of transcription from RNA polymerase II promoter	64
protein transport	61
intracellular protein transport	61
system development	61
neurological system process	56
cell cycle	53
response to stimulus	53
cell adhesion	49
proteolysis	47
intracellular signaling cascade	41
cell-cell signaling	41
ion transport	39
ectoderm development	39
protein modification process	39
vesicle-mediated transport	38
lipid metabolic process	36
nervous system development	36
mesoderm development	36
G-protein coupled receptor protein signaling pathway	34
apoptosis	33
cation transport	31
cell-cell adhesion	31
cellular component organization	30
synaptic transmission	27
immune response	26
carbohydrate metabolic process	23
cellular component morphogenesis	22
anatomical structure morphogenesis	22
protein amino acid phosphorylation	21

cell motion	19
mitosis	19
RNA metabolic process	16
sensory perception	16
endocytosis	15
phospholipid metabolic process	15
transmembrane receptor protein tyrosine kinase signaling pathway	14
reproduction	14
translation	13
response to stress	13
muscle contraction	13
exocytosis	13
induction of apoptosis	13
neurotransmitter secretion	13
mRNA processing	13
cytokine-mediated signaling pathway	11
cellular amino acid metabolic process	11
cellular amino acid and derivative metabolic process	11
DNA metabolic process	11
muscle organ development	11
gamete generation	11
respiratory electron transport chain	10
pattern specification process	10
angiogenesis	10
generation of precursor metabolites and energy	10
protein folding	10
MAPKKK cascade	10

Table 5 The ontological analysis of the genes modulated by the *WWOX* overexpression in SW480 cancer cell line. Pathways

**Pathway**	**Number of genes**
Unclassified	636
Wnt signaling pathway	13
Integrin signalling pathway	10
Inflammation mediated by chemokine and cytokine signaling pathway	10
PDGF signaling pathway	9
Huntington disease	8
Cadherin signaling pathway	8
Alzheimer disease-presenilin pathway	7
TGF-beta signaling pathway	7
Interleukin signaling pathway	6
Heterotrimeric G-protein signaling pathway-Gi alpha and Gs alpha mediated pathway	6
Axon guidance mediated by semaphorins	5
p53 pathway	5
p38 MAPK pathway	5

Table 6 The ontological analysis of the genes modulated by the *WWOX* overexpression in SW480 cancer cell line. Biological Process

**Biological Process**	**Number of genes**
metabolic process	287
primary metabolic process	276
Unclassified	233
cellular process	229
nucleobase, nucleoside, nucleotide and nucleic acid metabolic process	144
cell communication	140
signal transduction	136
protein metabolic process	106
transport	98
developmental process	89
transcription from RNA polymerase II promoter	89
transcription	89
regulation of transcription from RNA polymerase II promoter	70
immune system process	66
cell surface receptor linked signal transduction	59
protein transport	55
intracellular protein transport	55
system process	55
cellular component organization	53
cell cycle	50
intracellular signaling cascade	48
protein modification process	48
response to stimulus	43
system development	43
cell adhesion	42
neurological system process	42
cellular component morphogenesis	39
anatomical structure morphogenesis	39
proteolysis	39
mesoderm development	36
protein amino acid phosphorylation	33
vesicle-mediated transport	33
cell-cell signaling	27
lipid metabolic process	26
apoptosis	26
ectoderm development	25
nervous system development	22
DNA metabolic process	21
ion transport	20
carbohydrate metabolic process	20
G-protein coupled receptor protein signaling pathway	19
reproduction	19

cell-cell adhesion	18
RNA metabolic process	17
sensory perception	17
cation transport	16
immune response	16
gamete generation	16
translation	14
muscle contraction	14
cytokine-mediated signaling pathway	14
cell motion	14
organelle organization	14
mitosis	14
cellular defense response	13
endocytosis	13
visual perception	12
exocytosis	12
DNA replication	12
MAPKKK cascade	12
phosphate metabolic process	12
respiratory electron transport chain	10
generation of precursor metabolites and energy	10
establishment or maintenance of chromatin architecture	10
synaptic transmission	10

Table 7 The ontological analysis of the genes modulated by the *WWOX* overexpression in SW480 cancer cell line. Cellular component

**Cellular Component**	**Number of genes**
Unclassified	649
intracellular	47
cytoskeleton	38
extracellular matrix	26
extracellular region	26
actin cytoskeleton	18
microtubule	14
cytoplasm	8
mitochondrial inner membrane	7
mitochondrion	7
organelle	7
protein complex	5
intermediate filament cytoskeleton	4
cell junction	4
plasma membrane	4

Table 8 The ontological analysis of the genes modulated by the *WWOX* overexpression in SW480 cancer cell line.

**Molecular Function**	**Number of genes**
Unclassified	270
binding	242
catalytic activity	176
nucleic acid binding	140
protein binding	102
DNA binding	99
hydrolase activity	91
transcription factor activity	88
transcription regulator activity	88
receptor activity	61
transferase activity	58
structural molecule activity	56
enzyme regulator activity	42
structural constituent of cytoskeleton	38
kinase activity	32
transporter activity	31
hydrolase activity, acting on ester bonds	31
transmembrane transporter activity	31
protein kinase activity	27
receptor binding	26
peptidase activity	21
calcium ion binding	18
small GTPase regulator activity	16
cytoskeletal protein binding	15
enzyme inhibitor activity	14
GTPase activity	13
ligase activity	13
oxidoreductase activity	12
kinase regulator activity	11
phosphatase activity	11
RNA binding	11
G-protein coupled receptor activity	10
actin binding	10
